# Individual differences in the evolution of causal illusions

**DOI:** 10.1111/bjop.12754

**Published:** 2024-12-06

**Authors:** J. García‐Arch, J. Rodríguez‐Ferreiro, I. Barberia

**Affiliations:** ^1^ Dynamics of Memory Formation Group, Departament de Cognició, Desenvolupament i Psicologia de la Educació, Secció de Processos Cognitius, Institut de Neurociències (INUB) Universitat de Barcelona (UB) Barcelona Spain; ^2^ Grup de Recerca en Cognició i Llenguatge (GRECIL), Departament de Cognició, Desenvolupament i Psicologia de la Educació, Secció de Processos Cognitius, Institut de Neurociències (INUB) Universitat de Barcelona (UB) Barcelona Spain

**Keywords:** causal illusion, causal learning, contingency learning, illusion of causality, null contingency

## Abstract

In this research, we investigated individual differences in the formation and persistence of causal illusions. In a re‐analysis of existing data, we identified two clusters of participants – persistent and adjusting – based on their trajectories in learning from repeated exposure to null contingencies. The persistent cluster maintained stable causal illusions, while the adjusting cluster demonstrated a reduction over time. This re‐analysis provided a nuanced understanding of individual differences in causal learning, emphasizing the differential role of probability estimations in predicting causal judgements. These findings were replicated in a subsequent study, highlighting the robustness of the identified effects. In a pre‐registered study, we extended the paradigm to include a second phase (active phase) to assess how individual differences in causal illusion trajectories in the passive phase would manifest when participants had agency in the information gathering process. The results were consistent with those of the two previous studies and confirmed our primary hypothesis that the adjusting cluster would exhibit a lower tendency to introduce the candidate cause on learning trials, and would, therefore, observe a higher frequency of cause–absent trials. Together, these studies provide comprehensive insights into the underpinnings of causal illusion development and persistence, potentially informing de‐biasing interventions.

## BACKGROUND

Numerous studies have revealed that humans are prone to developing causal illusions, that is, misperceptions of the relationship between an event (candidate cause) and an outcome, despite their independent occurrence (see Matute et al., 2015, for a review). Researchers investigating these illusions in the laboratory often use a contingency learning task, in which participants are tasked with discerning an unconfirmed causal relationship between two events: a candidate cause and an outcome. One trial after the other, participants observe the state of each of the events (present or absent) in a hypothetical new occasion. The illusion occurs when, after a short learning experience in which both events occur independently of one another, participants indicate that the candidate cause is to some extent effective in producing the outcome. This is considered an ‘illusion’ since the expected normative response entails reporting the absence of a causal connection between the two events.

The ease with which these illusions can be formed in the laboratory is particularly concerning as it mimics the formation of erroneous causal beliefs in daily life (e.g. Chow et al., 2019; Matute et al., 2011). In fact, it has been observed that the intensity of the illusion that will be developed in a contingency learning task is predictive of the level of endorsement of superstitious, paranormal and pseudoscientific beliefs (Blanco et al., 2015; Griffiths et al., 2019; Torres et al., 2020, 2022; Vicente et al., 2023). This suggests that the way in which these illusions are artificially created in the laboratory may be a valid model of the way in which erroneous causal beliefs appear in real‐life situations (Chow et al., 2019).

Given the appropriate set of parameters, most participants develop causal illusions to some degree (e.g. Torres et al., 2020). A key factor that has been widely investigated is the ratio of occurrence of each event involved in the contingency learning task. In particular, when any or both the candidate cause and the outcome occur with a high probability (i.e. high cause and outcome densities), the tendency to connect them increases (Allan & Jenkins, 1983; Alloy & Abramson, 1979; Blanco et al., 2013; Chow et al., 2019; Hannah & Beneteau, 2009). An illustration of this set of parameters is shown in Table 1, which reflects the combined frequencies that we employed in a recent study (Torres et al., 2020). As can be seen, the experienced one‐way contingency (∆*P*, Allan, 1980) between the candidate cause and the outcome was null, as the difference in the probability of the outcome, *O*, in the presence and absence of the candidate cause, *C*, was zero [i.e. ∆*P* = *P*(*O*|*C*) − *P*(*O*|~*C*) = 0]. This involves that the proportion of occasions in which the outcome event occurred was the same whether the candidate cause was present [i.e. *P*(*O*|*C*) = *a*/(*a* + *b*) = 27/36 = .75] or absent [i.e. *P*(*O*|~*C*) = *c*/(*c* + *d*) = 9/12 = .75]. However, the outcome was present in a majority of trials (36 out of 48 cases, i.e. ‘*a*’ + ‘*c*’ cells in Table 1) and so was the candidate cause (36 out of 48 cases, i.e. ‘*a*’ + ‘*b*’ cells in Table 1). After observing all learning trials, participants in Torres et al. (2020) rated the causal relationship between the candidate cause and the outcome on a scale from zero (no relationship at all) to 100 (totally connected). Interestingly, only 3% of them gave ratings of zero, while 87% of the participants provided ratings equal or above the medium value of the scale (50), thus indicating the development of a generalized medium‐to‐strong causal illusion.

**TABLE 1:** Example of combined frequencies that typically lead to strong causal illusions.

	Frequency of cases in which the *outcome* is *present*	Frequency of cases in which the *outcome* is *absent*
Frequency of cases in which the *candidate cause* is *present*	27 (*a*)	9 (*b*)
Frequency of cases in which the *candidate cause* is *absent*	9 (*c*)	3 (*d*)

*Note:* Both the candidate cause and the outcome occur at a high rate: in 27 occasions, both the candidate cause and the outcome occurred together (cell *a*), in nine more cases the candidate cause was present without being followed by the outcome (cell *b*), in nine occasions the outcome occurred without being preceded by the candidate cause (cell *c*) and in just three cases neither the candidate cause nor the outcome occurred (cell *d*).

While the emergence of causal illusions is commonly observed under the adequate conditions, the intensity of these illusions vary significantly across individuals. Recently, researchers have devoted efforts to delineate these individual differences, as they might open the door to refine interventions aimed to ameliorate the formation or erroneous causal beliefs. For instance, individuals with a stronger tendency to interpret ambiguous events as occurrences of the outcome (Blanco et al., 2020) or those who tend to need less information before jumping to conclusions (Moreno‐Fernández et al., 2021) develop more intense causal illusions. Furthermore, some authors have proposed that participants might differ in the weight they attribute to the different pieces of information presented during the task. As pointed out by Griffiths et al. (2019), those who give a stronger relative weight to conjunctive information (i.e. occasions in which both the candidate cause and the outcome co‐occur, i.e. ‘a’ trials in Table 1), compared to disjunctive information (i.e. occasions in which only the candidate cause or the outcome occur, i.e. ‘b’ and ‘c’ trials in Table 1), might be more vulnerable to causal illusions. This argument is similar to that proposed by Moreno‐Fernández et al. (2021) to explain why individuals developing stronger causal illusions also tend to readily jump to conclusions in other tasks. According to Moreno et al., people more prone to causal illusions might show a stronger tendency to overweight confirmatory ‘a’ and ‘d’, compared to non‐confirmatory ‘b’ and ‘c’ trials. Likewise, Béghin et al. (2021; see also Béghin & Markovits, 2022) defended a dual strategy model, assuming that people can adopt two main strategies in the process of causal induction. Statistical reasoners would ‘tend to generate likelihood estimations of putative conclusions based on intuitive evaluations of readily accessible information concerning the judgment in question’ (Béghin et al., 2021, p. 472), whereas counter‐example reasoners, ‘focus more on a smaller amount of information and are particularly sensitive to potential counterexamples’ (Béghin et al., 2021, p. 472). Their participants were categorized as statistical or counter‐example reasoners with a strategy assessment task, and subsequently completed a contingency learning task. The results (see their study 2) in the contingency learning task were consistent with the idea that statistical reasoners' causal ratings were more affected by sufficiency information [i.e. the probability of the outcome among trials in which the candidate cause is present, i.e. *a*/(*a* + *b*) or *P*(*O*|*C*)] than those of counter‐example reasoners, which appeared to be mainly associated with necessity information [i.e. the probability of the outcome among trials in which the candidate cause is absent, i.e. *c*/(*c* + *d*) or *P*(*O*|~*C*)]. Counter‐example reasoners, with a stronger focus on necessity, would be more conservative when reaching their causal conclusions than statistical reasoners, concentrating on sufficiency information (Béghin & Markovits, 2022) and, in zero contingency tasks as the ones on which this study is focused, they would develop less intense causal illusions.

So far, the literature on causal illusions has primarily focused on identifying the factors that contribute to the development of these illusions (see Matute et al., 2015, for a review). Yet, a critical gap remains in our understanding of the progression and persistence of these illusions after prolonged exposure to the null contingency between the events in question. This information is crucial in determining whether these illusions are temporary, intermediate stages of learning or if they are stable or cumulative, which would necessitate societal efforts to de‐bias individuals and counter‐act the persistence of previously formed erroneous causal beliefs.

From this perspective, a recent work investigated whether extending the length of the learning phase in a contingency learning task would weaken or dissolve causal illusions (Barberia et al., 2019). In this work, the researchers used a contingency learning task with a cover story that has been widely employed in the literature (e.g. Blanco et al., 2013; Matute et al., 2011). Participants were asked to evaluate the effectiveness of an experimental drug as a treatment of a fictitious disease and were presented, in consecutive trials, with different fictitious patients experiencing the disease. Some of these patients were administered the experimental drug, while others were not, and the outcome regarding recovery varied among them. The length of training in the causal illusion literature usually fluctuates between 40 and 100 trials (e.g. Chow et al., 2019; Griffiths et al., 2019; Lovibond et al., 2023; Matute et al., 2011; Yarritu et al., 2014). Nevertheless, in this case it was extended to 288. These 288 trials were divided into six blocks of 48 randomly presented trials, which complied with the frequencies reflected in Table 1. Those frequencies were chosen to maximize the initial peak intensity of the illusion, with the aim of increasing the likelihood of observing any subsequent decrease. At test, participants were asked three questions. They first provided ‘causal ratings’, that is, their estimations of effectiveness of the experimental drug on a scale from 0 (*not effective*) to 100 (*totally effective*), where higher numbers indicate stronger causal illusions. They also estimated, out of 100 new patients, how many would overcome the crisis if they took the drug [*P*(*O*|*C*)_subj_] and if they did not [*P*(*O*|~*C*)_subj_]. One group of participants (henceforth *recurrent* group) responded to these three questions after every block of training (i.e. after observing 48 new fictitious patients), while the other group (henceforth *single* group) only answered these questions after completing the task.

The recurrent group showed a slight reduction in causal ratings from the first to the sixth block of trials. The estimated probability of recovery was higher when the drug was present [*P*(*O*|*C*)_subj_] than when it was absent [*P*(*O*|~*C*)_subj_]. Although *P*(*O*|~*C*)_subj_ increased over blocks, *P*(*O*|*C*)_subj_ remained unchanged. The single group's results after six blocks were similar to the recurrent group's after the first block, indicating that repeated evaluation, rather than merely more trials, influenced the evolution of causal illusions. In Barberia et al. (2019), the researchers proposed that one potential explanation for the impact of the recurrent rating periods on the illusion may be that by repeatedly asking participants to estimate both conditional probabilities, *P*(*O*|*C*)_subj_ and *P*(*O*|~*C*)_subj_, they indirectly educated them on the importance of considering both probabilities when evaluating a causal relationship. This repeated questioning may act as a de‐biasing factor, reducing a default underweighting of *P*(*O*|~*C*)_subj_ relative to *P*(*O*|*C*)_subj_.

Besides differential interpretation of received information, another key component that seems to affect the susceptibility to causal illusions is the search behaviour that participants exhibit when examining causal relationships. In order to investigate this possibility, researchers employ *active* forms of the contingency learning task. Here, participants can decide whether they want the candidate cause to be present or absent in each trial, and afterwards, they observe if the outcome occurs or not (whereas in the *passive* forms of the task, as the one by Torres et al. described earlier, participants are simply informed about both the state of the candidate cause and the outcome on each trial). In those active forms of the task, people who exhibit a more biased search behaviour, opting to have the candidate cause present in a substantial proportion of trials, tend to develop stronger causal illusions (Blanco et al., 2011). Note that, as indicated by Griffiths et al. (2018), search strategy in active contingency learning tasks could in fact be also indicative of differential weights given to different pieces of information, which would be expressed in search behaviour when given the option. This preference for specific types of information, such as conjunctive cases (in Griffiths et al.'s terms) or sufficiency information (in Béghin et al.'s terms) might produce a stronger tendency to introduce the candidate cause in active contingency learning tasks. Interestingly, this behavioural tendency also appears to be more marked in individuals endorsing unwarranted beliefs (Blanco et al., 2015; Torres et al., 2022).

In this work, we aimed to investigate individual differences in the evolution of causal illusions over repeated exposure and testing. Previous works indicate a modest overall decline in causal ratings (Barberia et al., 2019); however, our current research seeks to dissect the nuances of this decline. Specifically, we aim to determine whether the observed reduction represents a uniform diminution across all participants or if it reflects more complex patterns where certain individuals correct their misconceptions, while others either maintain or intensify their erroneous beliefs over successive trials (goal 1). We also aimed to determine if these potential differences in the evolution of causal illusions might be driven by a differential weighting of the two estimated conditional probabilities that are relevant for the contingency calculation (goal 2). More specifically, illusion correction might be a function of the importance given to each of the conditional probabilities. Our last goal (goal 3) was to determine whether participants' tendency to correct or persist in their causal illusions in a passive contingency learning task would predict their search behaviour in a subsequent active contingency learning context.

Guided by these research objectives, we first re‐analysed the data of the *recurrent* group from Barberia et al. (2019) utilizing longitudinal cluster analysis to delineate the potential individual differences in the evolution of causal illusions over time (see subsequent section). In order to strengthen the validity of our findings, we also conducted a replication study, confirming the key results and underscoring the robustness of our initial observations.^1^ Finally, we extended these results by means of a pre‐registered study with a nationally representative sample (UK) consisting of two phases. The first phase was a conceptual replication of the previous two studies. In the second phase, the procedure switched to an active one.

### Re‐analysis of Barberia et al. (2019)

In order to study potential individual differences between participants of the *recurrent* group in Barberia et al. (2019), we aimed to identify clusters of similar individual causal illusion trajectories over time. This approach goes beyond the aim of finding individual differences' predictors of causal illusions and aims to characterize individuals' differential behaviour with regards to the phenomenon itself. To that end, we modelled cluster trajectories by means of *k*‐means longitudinal clustering with the R package ‘kml’ (Genolini & Falissard, 2016). Modelling individual trajectories through *k*‐means longitudinal (KML) clustering hill‐climbing algorithms provides a flexible and distribution free approach to group individuals according to their behaviour across time (Genolini & Falissard, 2011), which has proven to be a useful strategy in a wide variety of clinical fields (Hall et al., 2017; Panlilio et al., 2020; Wood et al., 2021). In contrast to traditional *k*‐means clustering, which works on static data, KML applies clustering on trajectories – identifying groups of individuals that follow similar time‐evolution patterns. The KML algorithm systematically explores a range of cluster counts to identify the best configuration. This process is guided by empirical data using robust non‐parametric quality criteria such as the Calinski and Harabatz criterion. This criterion assesses the ratio of between‐cluster variance to within‐cluster variance, maximizing the formula *C*(*k*) = (trace(*B*)/trace(*W*)) (*n* − *k*/*k* − 1), where *B* denotes the between‐ and *W* within‐cluster sum of squares, respectively, and *n* is the total number of observations. The algorithm starts by assigning individuals (based on their longitudinal trajectories) to clusters randomly. Each cluster is represented by a centroid, which, in the case of longitudinal data, is a ‘group trajectory’ representing the average trajectory of the individuals assigned to that cluster. Random initialization can sometimes result in poor solutions. By performing *n* permutations (or iterations), KML reduces the risk of arriving at a sub‐optimal clustering solution that might happen due to an unfortunate starting point. After running these multiple permutations, the algorithm selects the best clustering solution based on an objective criterion, such as the Calinski and Harabasz index (see before). Each permutation undergoes the iterative process of re‐calculating centroids, re‐assigning individuals and updating cluster memberships until the solution converges (i.e. no further changes can be made). After the convergence of each permutation, the resulting clusters are evaluated. Once all 100 permutations are complete, the algorithm compares the results from all permutations and selects the clustering solution that yields the best fit to the data, typically the solution with the highest Calinski–Harabasz score. In our study, we allowed algorithm to run 100 permutations and try partitions up to six clusters.

**FIGURE 1:**
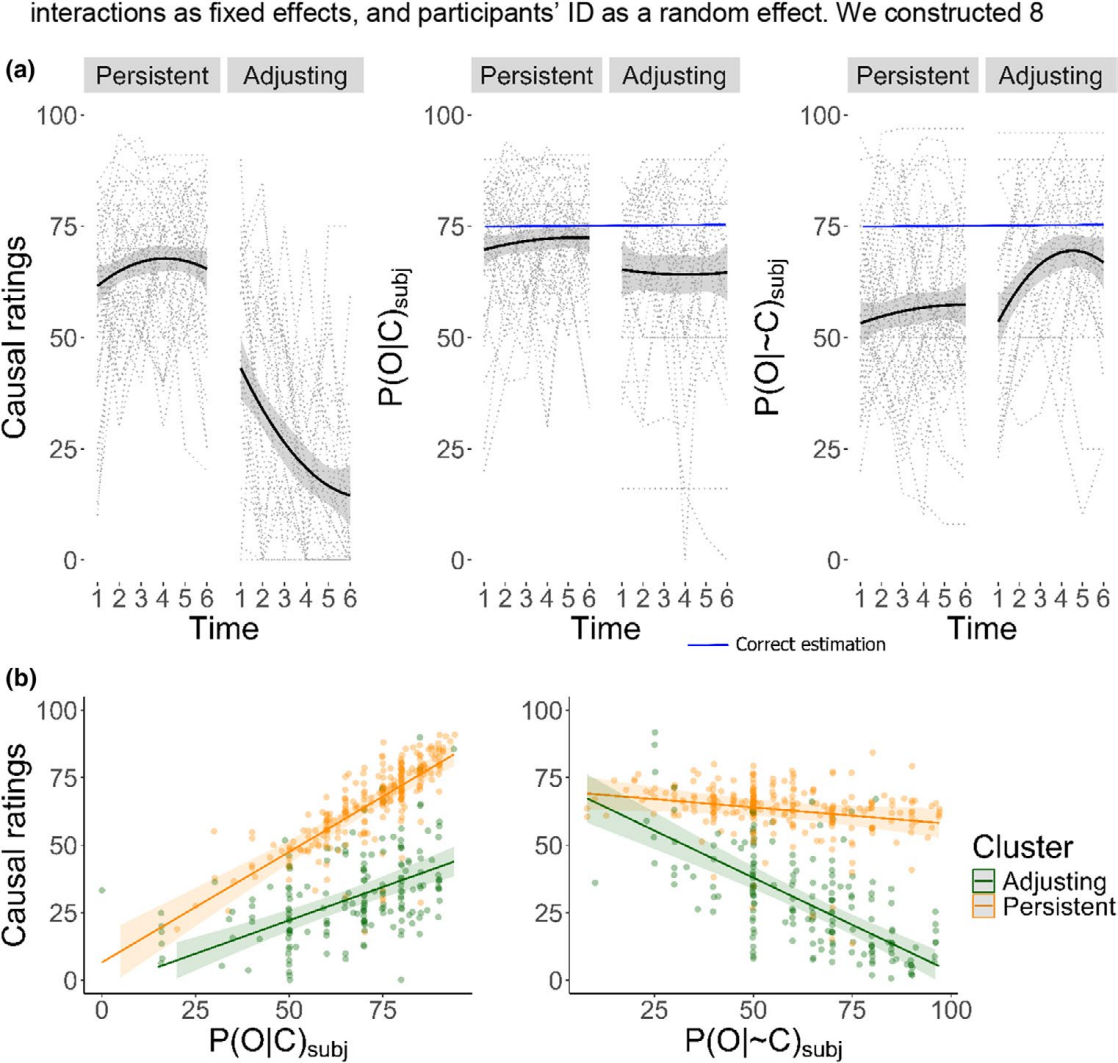
Distribution of causal illusion ratings, *P*(*O*|*C*)_subj_ (*B*), and *P*(*O*|~*C*)_subj_ over time as a function of cluster membership (panel a). Relationship of *P*(*O*|*C*)_subj_ (a) and *P*(*O*|~*C*)_subj_ (b) with causal ratings by cluster membership (panel b).

The KML algorithm found two clusters as the best possible partition. Of the participants, 58.7% were identified as belonging to cluster A and 41.3% to cluster B. We found that cluster A was characterized by a stable pattern of causal ratings, suggesting no change across time. Cluster B showed a decrease in their causal illusions across time. For simplification, cluster A will be called the *persistent* cluster, and cluster B will be named the *adjusting* cluster henceforth (see Figure 1, panel a). To test the observed differences, we conducted a cluster by time mixed ANOVA on causal ratings. Results showed a significant main effect of cluster, *F*(1, 73) = 208.991, *p* < .001, *η* ^2^ = 0.737, 90% CI [0.728, 0.739], a significant main effect of time *F*(3.64, 265.63) = 6.595, *p* < .001, *η* ^2^ = 0.070, 90% CI [0.069, 0.076] and a significant cluster × time interaction, *F*(3.64, 265.63) = 11.823, *p* < .001, *η* ^2^ = 0.127, 90% CI [0.125, 0.134]. *Post‐hoc* pairwise comparisons (Tukey‐adjusted *p* values; estimated marginal means *emmeans*, Lenth, 2020) revealed that participants in the *adjusting* cluster displayed a significantly less intense causal illusion in all time points than those in the *persistent* cluster (see Table S1). Cluster × time interaction suggested a progressive increase in differences between groups. We performed a polynomial trend contrast in each group. For the persistent cluster, there was no significant variation in causal ratings over time (all *p*s > .883). In contrast, the adjusting cluster showed a significant linear decrease in causal illusions (*p* < .001).

After identifying differences in cluster trajectories, we examined whether clusters differed in their estimations of conditional probabilities (Figure 1). Two separate mixed ANOVAs were conducted for each estimation. Results indicated significant differences between clusters in *P*(*O*|*C*)_subj_, with higher values in the persistent cluster, but no significant effects for time or cluster × time interaction. For *P*(*O*|~*C*)_subj_, there was a significant main effect of cluster and time, but no significant cluster × time interaction (see Supporting Information).

To go beyond cluster characterization, we tested whether the predictive capacity of *P*(*O*|*C*)_subj_ and *P*(*O*|~*C*)_subj_ over the causal ratings was different depending on cluster membership (and time), which would provide additional information on the potential differential underlying factors driving causal ratings in each cluster. Consequently, we built a linear mixed effects model with causal rating as the dependent variable, cluster, time, *P*(*O*|*C*)_subj_, *P*(*O*|~*C*)_subj_, and all cluster two‐way interactions as fixed effects, and participants' ID as a random effect. We constructed eight additional models to test if any of them accounted for causal ratings better than the proposed model. We compared the fitted models by means of the Bayesian information criteria index (BIC) which penalizes model complexity (Neath & Cavanaugh, 2012). BIC determined that the best model was the one including main effects for cluster, time, *P*(*O*|*C*)_subj_, *P*(*O*|~*C*)_subj_, and all cluster two‐way interactions (BIC: 3728.359). The list of the models and their associated BIC indices are reported in Table S2. Global tests resulted significant for all model parameters (see, Table S3). Of primary interest, we decomposed both target interactions *P*(*O*|*C*)_subj_ × cluster and *P*(*O*|~*C*)_subj_ × cluster through the estimation of marginal means of their linear trends (Lenth, 2020). *Post‐hoc* analysis showed that both clusters displayed a negative relationship between *P*(*O*|~*C*)_subj_ and causal ratings, and that the interaction effect cluster × *P*(*O*|~*C*)_subj_ was due to a greater negative slope in the *adjusting* as compared to the *persistent* cluster (persistent cluster: *β* = −0.122, *SE* = 0.056, 95% CI [−0.234, −0.011]; adjusting cluster: *β* = −0.698, *SE* = 0.073, 95% CI [−0.842, −0.554]). *Post‐hoc* analysis also revealed that both clusters displayed a positive relationship between *P*(*O*|*C*)_subj_ and causal ratings, and that the interaction effect cluster × *P*(*O*|*C*)_subj_ was due to a greater positive slope in the *persistent* as compared to the *adjusting* cluster (persistent cluster: *β* = .819, *SE* = 0.071, 95% CI [0.679, 0.959]; adjusting cluster: *β* = 0.494, *SE* = 0.068, 95% CI [0.360, 0.629]); see Figure 1 (panel b).

So far, results indicated that cluster differential causal ratings were a function of cluster differential use of subjective probabilities [*P*(*O*|*C*)_subj_ and *P*(*O*|~*C*)_subj_]. The resulting model, however, did not include the possibility that clusters differed in the combined use of both probabilities (i.e. the difference between them, i.e. the subjective contingency or Δ*P*_subj_ value). We, therefore, tested if a model including the difference between *P*(*O*|*C*)_subj_ and *P*(*O*|~*C*)_subj_, instead of *P*(*O*|*C*)_subj_ and *P*(*O*|~*C*)_subj_ separately, would improve the current model's fit. Results showed that the model including separate components for *P*(*O*|*C*)_subj_ and *P*(*O*|~*C*)_subj_ outperformed the new model tested (BIC1: 3728.359; BIC2: 3768.961 respectively).

### Discussion

The re‐analysis of Barberia et al. (2019) revealed the existence of two clusters of participants with distinct intensity and evolution of causal illusions. The differential trajectories observed in each group suggested that while one group persisted in their causal illusion (*persistent* cluster), the other group showed a progressive attenuation (*adjusting* cluster). Crucially, causal ratings were found to be more strongly associated with the estimated *P*(*O*|~*C*)_subj_ for adjusting participants than for the persistent ones, while the opposite was true regarding the estimated *P*(*O*|*C*)_subj_. The pattern of results was corroborated in a subsequent replication study presented as Supporting Information. In short, in this replication study the longitudinal cluster algorithm corroborated the presence of two distinct clusters – ‘persistent’ and ‘adjusting’ – with 70.7% of participants in the persistent cluster and 29.3% in the adjusting cluster. These clusters exhibited differential trajectories in the evolution of causal illusions. Specifically, the persistent cluster maintained stable causal illusions over time, while the adjusting cluster demonstrated a significant reduction, showcasing a linear decrease in illusions as blocks progressed. Consistent with our initial findings, we also found significant differences between clusters in their *P*(*O*|~*C*)_subj_, with the adjusting cluster consistently providing higher estimations than the persistent cluster. No significant differences between clusters were found for *P*(*O*|*C*)_subj_ in the replication study. We also tested the robustness of our initial model by examining its ability to predict causal ratings based on clusters, time and probability estimates [*P*(*O*|*C*)_subj_ and *P*(*O*|~*C*)_subj_]. Using a linear mixed effects model, we fully replicated the model where both probability estimates significantly interacted with cluster membership to influence causal ratings. The replication study robustly confirmed the reliability of our data‐driven cluster analysis by consistently identifying similar clusters with parallel trajectories in causal illusion formation across different datasets. Additionally, the study reinforced the significant role of probability estimates in influencing these trajectories, further underscoring the method's robustness and its applicative value in understanding individual differences in the development of causal illusions.

### Pre‐registered study

Building on the insights gained from the re‐analysis of Barberia et al. (2019) and its subsequent replication (see Replication Study, Supporting Information), we decided to run a final study whose sample size, design, hypotheses and analysis plan were pre‐registered (https://aspredicted.org/hd56s.pdf). We ensured that the sample size of the study was big enough to be able to detect the relevant effects observed in our previous studies. Moreover, we gathered a representative sample (UK population), instead of relying on a sample exclusively composed by (Spanish) psychology students, which allowed us to better characterize the prevalence of adjusting and persistent tendencies in the general population.

The goal of the study was twofold. First, it sought to corroborate our previous results and second, to expand the understanding of individual differences in causal illusions trajectories by introducing an active component to the previously passive contingency learning task. To this end, the contingency learning task included both a passive phase which was similar to that of our previous studies, as well as a novel active phase in which participants had the chance to control the presence or absence of a potential cause to learn how it affected the outcome. Regarding the passive phase, first (hypothesis 1) we expected the emergence of two clusters of participants, one of them persisting in their causal illusions over blocks of testing (persistent cluster) and the other diminishing their illusions from the first to the last block of trials (adjusting cluster). Second (hypothesis 2), we predicted that the persistent cluster would show a higher *P*(*O*|*C*)_subj_ than the adjusting cluster, and the opposite would be true for the *P*(*O*|~*C*)_subj_. Third (hypothesis 3), we anticipated that the predictive capacity of the *P*(*O*|*C*)_subj_ over causal ratings would be higher for persistent than for adjusting participants, whereas the opposite would be true regarding the predictive capacity of the *P*(*O*|~*C*)_subj_.

The new active phase allowed us to explore how individual differences in the evolution of causal illusions in a passive context would manifest when participants are granted agency in the information accumulation process. In this situation, the usual strategy involves a larger exposure to the *P*(*O*|*C*), that is, patients taking the drug in our scenario, than to the *P*(*O*|~*C*), that is, patients not taking it (see, e.g. Barberia et al., 2013; Blanco et al., 2011), while the most adequate would be a balanced sampling of both types of patients (Barberia et al., 2013). However, note that, in our case (after having observed a majority of patients taking the drug during the passive phase, see Table 1), it could even be considered that the most efficient strategy might be to preferably search for patients that did not take the drug when given the option (in the active phase). In any case, independently of the absolute exposure to the drug shown by the participants, we hypothesized that the adjusting cluster, which appears to attribute a higher importance to the *P*(*O*|~*C*) than the persistent one, would show a reduced tendency to administer the drug (hypothesis 4).

## METHOD

### Participants

We conducted a conservative power analysis to determine the required sample size for this study. With the goal of replicating the main finding from the re‐analysis of Barberia et al. (2019) and the replication study (Supporting Information), the power analysis determined that in order to detect an effect of *η* ^2^ = 0.03 with a correlation between repeated measures of .3, we would need at least 76 participants. Note that this is a conservative power analysis aiming to capture the cluster by time interaction, which effect sizes were *η* ^2^ = 0.127 in the re‐analysis, and *η* ^2^ = 0.068 in its subsequent replication. We also computed the required sample size to test whether participants in the *persistent* cluster would also display a higher tendency than participants in the *adjusting* cluster to administer the drug in the active phase, that is, a between‐subjects *t* test comparing the proportion of trials in which the drug was administered, *P*(*C*). We decided to set a rather conservative effect size (*d* = 0.35, two‐tailed), which requires a minimum of 260 participants. However, we increased the planned sample size to 300, which is the minimum required for the recruitment of a representative sample (sex, age and ethnicity among the UK residents) in prolific (154 females, *M*_age_ = 46.21, *SD*_age_ = 15.25). All participants provided informed consent. The ethics committee of the University of Barcelona (Institutional Review Board IRB00003099) approved the study protocols.

### Procedure

We adapted the contingency learning task from Barberia et al. (2019) to this study (the experimental task, implemented in Qualtrics, can be found at https://osf.io/tmc48/?view_only=69879b34715b453b8187a2042261c51c and the initial written instructions for the task can be found at Supporting Information). The contingency learning task comprised four blocks, and combined passive and active learning phases. The initial three blocks involved a passive learning task. Participants were presented with a series of fictitious medical cases, each depicting a patient experiencing a crisis of the *Lindsay Syndrome*, an allegedly rare and dangerous disease. Participants were informed about an experimental drug, Batatrim, which was posited as a potential cure for the crises produced by the syndrome. During the task, they encountered scenarios such as ‘This patient is experiencing a crisis and receives BATATRIM’ or ‘This patient is experiencing a crisis and does not receive anything’. Following each scenario, participants were asked to predict whether the patient would overcome the crisis. Following their prediction, they received feedback indicating whether the patient did indeed overcome the crisis or not. Each block consisted of 48 trials. The frequencies of the different types of trials within each block were those reflected in Table 1, that is, 27 patients that recovered after receiving the drug, nine patients which were not cured after receiving the drug, nine patients that recovered without receiving the drug and three patients that neither received the drug nor recovered from the crisis.

The fourth block of our study marked a transition to an active learning phase, where participants' roles shifted from observers to decision makers. In this phase, they were presented with 48 new fictitious patients, each experiencing a crisis due to *Lindsay Syndrome*. Unlike the passive blocks, participants were now given the critical choice of whether to administer the experimental drug Batatrim to each patient. Specifically, they were told that they would observe some more patients experiencing a Lindsay Syndrome crisis, but that now they would be deciding whether they wanted to administer Batatrim or not to each of them. For each patient, after they made their choice (by clicking ‘administer’ or ‘do not administer’ buttons), they were shown whether the patient overcame the crisis. The outcomes of the trials were pre‐determined, that is, irrespective of the participants' choices to administer or withhold Batatrim, 36 patients were programmed to recover and 12 were not, in random order for each participant.

Following each of the four blocks of trials, participants engaged in a series of judgement tasks, which paralleled those employed by Barberia et al. (2019). Participants were asked to evaluate the effectiveness of Batatrim and to make probabilistic predictions about patient outcomes under different treatment scenarios. Participants were randomly assigned to one of four possible sequences for these judgement tasks. These sequences determined the order in which they were asked about the effectiveness of the drug (i.e. causal rating, ‘To what extent do you think that Batatrim is effective at overcoming the crises produced by Lindsay Syndrome?’, which was answered in a scale from 0, ‘not effective at all’ to 100, ‘totally effective’), the probability of overcoming the crisis when the drug was administered [i.e. *P*(*O*|*C*)_subj_, ‘Imagine 100 NEW PATIENTS that are experiencing a crisis produced by the Lindsay Syndrome and TAKE BATATRIM. How many do you think will overcome the crisis?’], and the probability of overcoming the crisis without any treatment [i.e. *P*(*O*|~*C*)_subj_, ‘Imagine 100 NEW PATIENTS that are experiencing a crisis produced by the Lindsay Syndrome and DO NOT TAKE ANYTHING. How many do you think will overcome the crisis?’]. The four possible orders were: (1) causal rating, *P*(*O*|*C*)_subj_, *P*(*O*|~*C*)_subj_; (2) causal rating, *P*(*O*|~*C*)_subj_, *P*(*O*|*C*)_subj_; (3) *P*(*O*|*C*)_subj_, *P*(*O*|~*C*)_subj_, causal rating; (4) *P*(*O*|~*C*)_subj_, *P*(*O*|*C*)_subj_, causal rating. The randomly assigned order for each participant was maintained across all four evaluation blocks.

## RESULTS

**FIGURE 2:**
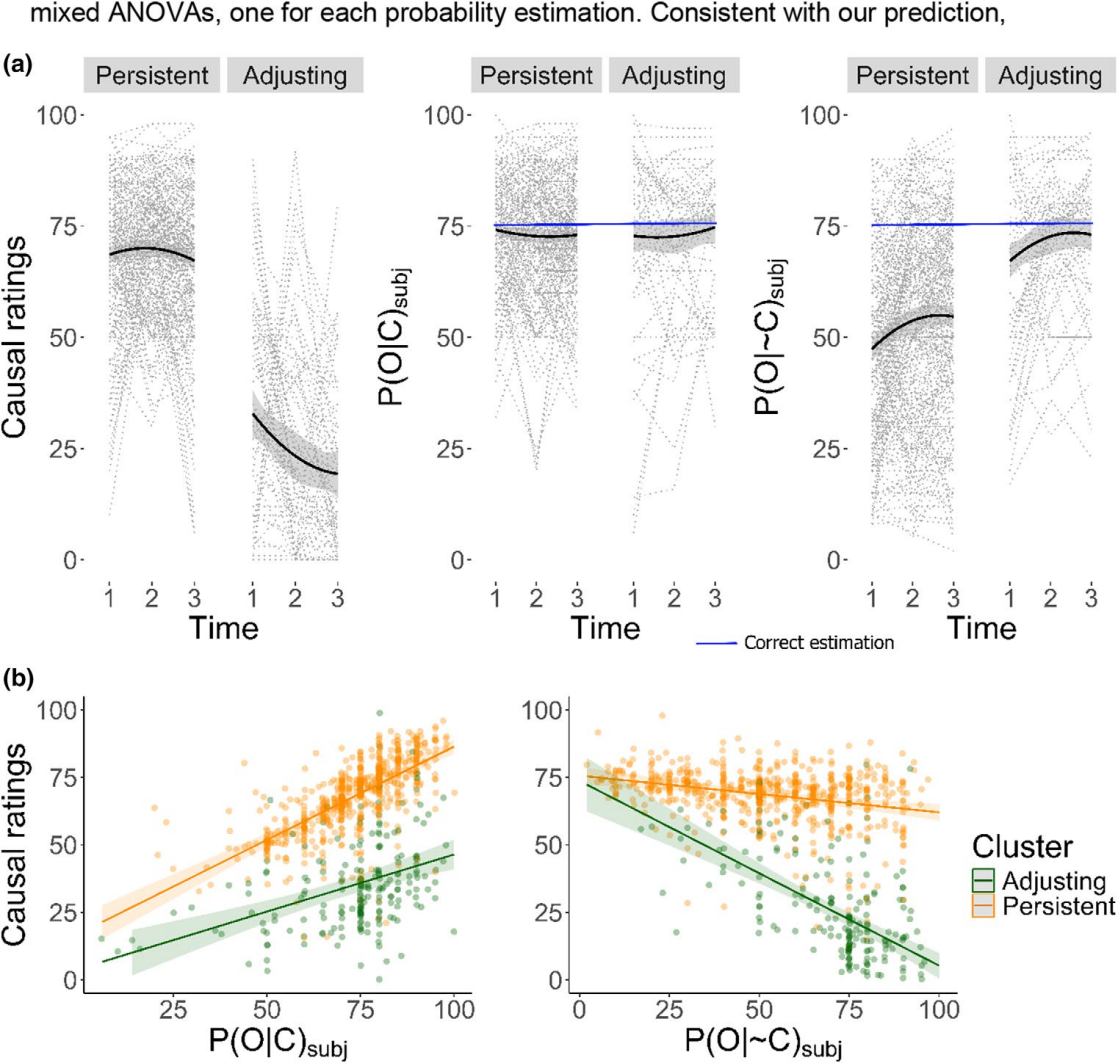
Distribution of causal ratings, *P*(*O*|*C*)_subj_ (B), and *P*(*O*|~*C*)_subj_ over time as a function of cluster membership in our pre‐registered study (panel a). Relationship of *P*(*O*|*C*)_subj_ (a) and *P*(*O*|~*C*)_subj_ (b) with causal ratings by cluster membership (panel b).

Consistent with our hypothesis 1, the KML algorithm found two clusters as the best possible partition. Of the participants, 77% were identified as belonging to cluster A and 23% to cluster B. In line with our previous results, cluster A demonstrated a consistent trend in causal ratings, indicating no variation over time (persistent cluster). Conversely, cluster B (adjusting cluster) exhibited a reduction in causal illusions over time (Figure 2, panel a).

To test the observed differences, we run a cluster by time mixed ANOVA on causal ratings. Results showed a significant main effect of cluster, *F*(1, 298) = 654.251, *p* < .001, *η* ^2^ = 0.687, 90% CI [0.682, 0.688], a significant main effect of time, *F*(1.834, 546.586) = 13.663, *p* < .001, *η* ^2^ = 0.044, 90% CI [0.04, 0.044], and a significant cluster × time interaction, *F*(1.834, 546.586) = 11.058, *p* < .001, *η* ^2^ = 0.036, 90% CI [0.032, 0.036]. *Post‐hoc* analyses replicated the findings from the re‐analysis of Barberia et al. (2019) (see Supporting Information).

**FIGURE 3:**
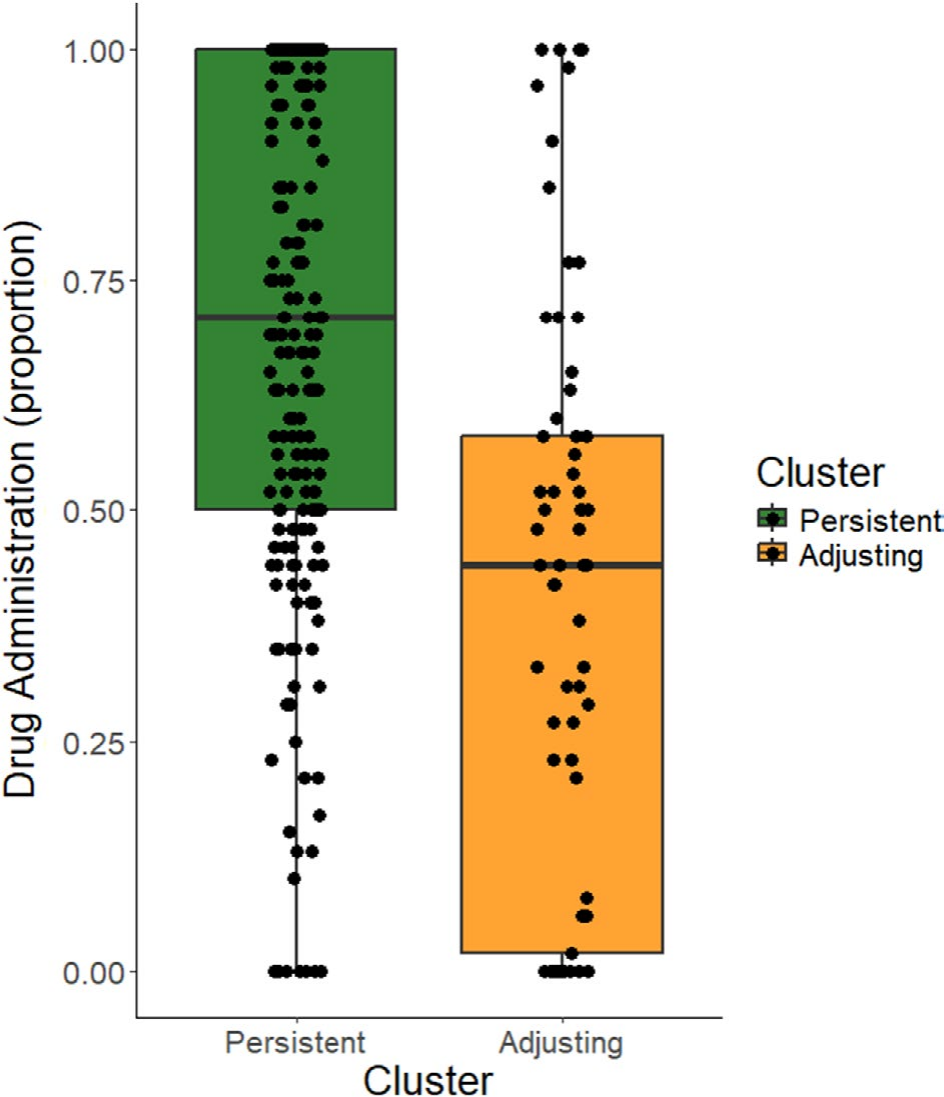
Differences in drug administration (proportion) between clusters in the pre‐registered study.

Subsequently (hypothesis 2), we analysed whether clusters also differed in their estimations of conditional probabilities (Figure 2, panel a). We conducted two separate mixed ANOVAs, one for each probability estimation. Consistent with our prediction, results regarding *P*(*O*|~*C*) _subj_ showed a significant main effect of cluster, *F*(1, 298) = 63.131, *p* < .001, *η* ^2^ = 0.172, 90% CI [0.169, 0.178], a significant main effect of time, *F*(1.697, 505.605) = 17.076, *p* < .001, *η* ^2^ = 0.051, 90% CI [0.05, 0.055], and no significant cluster × time interaction, *F*(1.697, 505.605) = 0.153, *p* = .824, *η* ^2^ < 0.001, 90% CI [0, 0.001] (Figure 3). *Post‐hoc* contrast revealed that the *persistent* cluster provided a significantly lower *P*(*O*|~*C*)_subj_ than the *adjusting* cluster (*M* = −19.044, *SE* = 2.396, *t*(298) = −7.946, *p* < .001). *Post‐hoc* analysis of the time effect revealed that the *P*(*O*|~*C*)_subj_ was lower in Time 1 with respect to Time 2 and Time 3 [*M* = −5.979, *SE* = 1.329, *t*(298) = −4.5, *p* < .001; *M* = −6.595, *SE* = 1.410, *t*(298) = −4.678, *p* < .001]. No significant differences were found between Time 2 and Time 3 [*M* = −0.616, *SE* = 0.954, *t*(298) = −0.646, *p* = .794]. Contrary to our prediction, but consistent with the replication study, results regarding *P*(*O*|*C*)_subj_ returned no significant differences between clusters *F*(1, 298) = 0.004, *p =* .948, *η* ^2^ = 0, 90 CI[0, <.001]. Moreover, no significant effects were found for time, *F*(1.955, 582.727) = 0.788, *p* = .453, *η* ^2^ = 0.003, 90% CI [0, 0.001], or cluster × time interaction, *F*(1.955, 582.727) = 1.285, *p* = .277, *η* ^2^ = 0.004, 90% CI [0.002, 0.003] (Figure 2a).

Next (hypothesis 3), we tested whether the predictive capacity of the *P*(*O*|*C*)_subj_ and *P*(*O*|~*C*)_subj_ over causal ratings was different between clusters (and time). We compared the same set of mixed effects models employed in the replication study and compared them using the BIC. The best‐fitting model replicated the previously obtained results (i.e. causal rating ~ [time + *P*(*O*|*C*)_subj_ + *P*(*O*|~*C*)_subj_] × cluster + [1|participant]). Global tests on model predictors returned statistically significant effects for all model parameters (all *p*s < .013). We subsequently decomposed both target interactions *P*(*O*|*C*)_subj_ × cluster and *P*(*O*|~*C*)_subj_ × cluster to explore whether results matched those obtained before. *Post‐hoc* analyses showed that both clusters displayed a negative relationship between *P*(*O*|~*C*)_subj_ and causal ratings, and that the interaction effect cluster × *P*(*O*|~*C*)_subj_ was due to a greater negative slope in the *adjusting* cluster as compared to the *persistent* cluster (*persistent*: *β* = −0.136, *SE* = 0.028, 95% CI [−0.192, −0.079], *adjusting*: *β* = −0.684, *SE* = 0.071, 95% CI [−0.823, −0.544]). *Post‐hoc* analyses also revealed that both clusters displayed a positive relationship between *P*(*O*|*C*)_subj_ and causal ratings and that the interaction effect cluster × *P*(*O*|*C*)_subj_ was due to a greater positive slope in the *persistent* cluster as compared the *adjusting* cluster (*persistent*: *β* = .691, *SE* = 0.045, 95% CI [0.601, 0.781], *adjusting*: *β* = .423, *SE* = 0.073, 95% CI [0.279, 0.567]), which fully replicated the results obtained in both the re‐analysis and the replication studies (Figure 2, panel b).

Finally (hypothesis 4), we aimed to test whether participants belonging to the persistent and adjusting clusters would differ in their percentage of administration of the drug during the active block of the null contingency task. To that end, we computed the percentage of times each participant administered the drug during the block (see Figure 3) and compared this measure between clusters by means of an independent samples *t* test. In line with our prediction, results indicated that the persistent cluster administered the drug with a higher frequency than the adjusting cluster (persistent: *M* = 70.571%, *SD* = 27.909, adjusting = 39.971%, *SD* = 32.606, *t*(298) = 7.687, *p* < .001, *d* = 1.01).

Following the suggestion of a reviewer, for the three experiments reported, we explored whether cluster membership was associated with the order in which the causal and conditional probabilities' questions were answered. Note that about half of the participants first estimated the two conditional probabilities of recovery (in the presence and absence of the experimental drug) and after provided their causal ratings, while this order was reversed for the rest of them. Our analyses indicated that there was no association between cluster membership and whether the causal rating was provided before or after estimating the conditional probabilities of recovery, *X* ^2^(1) = 0.001, *p* = .922, *X* ^2^(1) = 0.611, *p* = .434, and *X* ^2^(1) = 2.059, *p* = .151, for the re‐analysis, the replication and the pre‐registered study respectively. This suggests that cluster membership cannot be reduced to an order effect, by which previous estimations of conditional probabilities might produce an adjustment in the subsequent causal rating.

## GENERAL DISCUSSION

This work aimed to investigate individual differences in the intensity and persistence of causal illusions. It did so both through the re‐analysis of data from Barberia et al. (2019) and the analysis of new data collected with this aim. Utilizing longitudinal cluster modelling, we identified two distinct clusters of participants. The first one, characterized as the ‘persistent’ cluster, displayed stronger causal illusions in the initial testing period and did not show any adjustment to the real null contingency in subsequent testing periods. In contrast, the second cluster, referred to as ‘adjusting’, exhibited lower causal illusions in the initial testing period and a subsequent gradual reduction of their causal illusions over exposure to subsequent testing blocks. The identification of two differentiated clusters of participants illuminates the important impact of individual differences on the development and persistence of causal illusions, providing novel insights for precision interventions tailored to the cognitive profiles observed, which might be crucial for effectively correcting erroneous causal inferences in educational and clinical settings.

We found that the persistent and adjusting cluster consistently differed both in their estimation of the base rate of the outcome and in the predictive value of the estimated conditional probabilities of the outcome over their causal ratings. Specifically, while the estimation of the probability of the outcome in the presence of the candidate cause, *P*(*O*|*C*)_subj_, did not vary over time and overall was similar between clusters (significant differences only emerged in the re‐analysis of Barberia et al., but neither in the subsequent replication nor in the final pre‐registered study), estimations of the probability of the outcome in the absence of the candidate cause, *P*(*O*|~*C*)_subj_, increased over blocks in both clusters, but were consistently lower in the persistent cluster compared to the adjusting one. Crucially, the differences in the strength of causal illusions between the two clusters appeared to be more complex than simple variations in the *P*(*O*|~*C*)_subj_. A notable difference was observed in the predictive capacity of *P*(*O*|*C*)_subj_ and *P*(*O*|~*C*)_subj_ over causal judgements as a function of cluster membership. The *P*(*O*|*C*)_subj_ had a higher predictive power for causal judgements in the persistent group, while the opposite pattern was observed for the *P*(*O*|~*C*)_subj_. This estimate had a higher predictive power for causal judgements in the adjusting group and a lower predictive capacity in the persistent group.

Moreover, this work not only investigated individual differences when contingency learning occurs after passively receiving information, but also the relationship between this passive learning and active strategies that operate when the learner is given the option to decide how to search for further information. The findings align with the notion that there is a prevalent inclination within the persistent cluster to predominantly consider instances where the candidate cause co‐occurs with the outcome, whereas the adjusting cluster demonstrates a contrasting propensity to focus on scenarios where the outcome occurs independently of the cause. Thus, the active phase of our last experiment demonstrated that, when free to do so, persistent participants tended to prefer to introduce the target cause in more occasions than the adjusting ones did. In fact, the adjusting cluster introduced the target cause in 40% of the trials, while the persistent cluster presented this target cause in about 70% of the trials. Note that, given that the number of participants being included in this latter cluster was more than double the number assigned to the adjusting one, if all the participants were simply analysed together, a general mean tendency to introduce the target case in more than half of the trials would emerge, *M* = 63.53, *SD* = 31.73, *t*(299) = 7.385, *p* < .001, *d* = 0.43, consistent with that observed in previous studies (Barberia et al., 2013, 2018; Blanco et al., 2011). Our cluster approach suggests that, even though the preference for cause–present cases seems dominant, some people might indeed tend to introduce the target cause in a relatively low amount of trials, and these people seem to be the ones especially considering the *P*(*O*|~*C*) when learning passively.

Prior research has suggested that people might differentially weight the available pieces of information, resulting in varying degrees of susceptibility to causal illusions (e.g. Griffiths et al., 2019; Moreno‐Fernández et al., 2021). Moreover, unbalanced weighting of different types of evidence might operate at different levels. For instance, as noted by Perales and Shanks (2007), one may differentially encode each piece of information during learning due to memory or attentional factors or assign them different degrees of relevance when using them to make the judgement (or even integrate them sub‐optimally due to computational errors during that same phase, see Mandel & Lehman, 1998). Our findings indicate that individual differences regarding information weighting might impact at least three intertwined levels of causal learning and inference.

First, weighting might affect the processing of the specific cell frequencies (see Table 1) or conditional probabilities during training, producing tendencies to overestimate or underestimate the experienced *P*(*O*|*C*) and/or *P*(*O*|~*C*). In this sense, in our experiments, those participants ameliorating their causal ratings along training also gave *P*(*O*|~*C*) estimations that were higher and closer to the *P*(*O*|*C*). In contrast, persistent individuals could be characterized by a bias in encoding, expressed as an underestimation of the probability of the outcome in the absence of the target cause.

Second, differential weighting could express at a decisional stage, when participants have to decide how to integrate acquired correlational information in order to reach a causal conclusion. To this respect, in the present experiments causal impressions of those participants adjusting their illusions over time were more associated with their own *P*(*O*|~*C*) estimation, compared to those participants that did not correct over time. Persistent participants, in contrast, presented a bias in integration, manifested as a poorer ability to incorporate the base‐rate information when having to decide the extent to which the candidate cause was connected to the outcome.

Third, unequal weighting might also impact the decisional process during the information gathering phase by favouring information search strategies that focus to varying extents on cause–present trials. In our experiments, persistent participants who tended to both underestimate the experienced *P*(*O*|~*C*) and provide causal ratings that were less dependent of it in a passive scenario also showed an increased tendency to introduce the candidate cause when they switched to an active information‐searching stage. In other words, they showed a search bias, focusing on instances where the candidate cause was present.

Our results are consistent with the observations by Béghin et al. (2021). Based on a strategy assessment task, they classified their participants as either statistical or counter‐example reasoners, who subsequently appeared to respectively focus more on sufficiency [i.e. *P*(*O*|*C*), thus mapping onto our persistent participants] or necessity [i.e. *P*(*O*|~*C*), thus mapping onto our adjusting participants] information. In contrast, in our study we employed data‐driven longitudinal modelling to obtain groups of participants based on their causal rating trajectories. Moreover, as indicated in previous paragraphs, differences among groups could be explained by differential weights given to different pieces of evidence gathered during the contingency learning process, without the need of assuming qualitative differences based on differential underlying processes. In any case, it might be worth confirming whether our persisting cluster aligns with statistical reasoners as described in previous work, while the adjusting cluster fits the qualitative profile of counter‐example reasoners.

The relation between our results and other accounts of the emergence of causal illusions such as those outlined by Griffiths et al. (2018) or Moreno‐Fernández et al. (2021) is less straightforward because their predictions focus on information related to specific trial types, either conjunctive or confirmatory such as ‘a’ trials (see Table 1), instead of conditional probabilities. In any case, the emphasis on the association between overweighting of cause–outcome co‐occurrences and causal illusion seems to go in line with our observation that participants preferentially focusing on *P*(*O*|*C*) develop stronger illusions.

It is worth noting that we have favoured a specific causal direction regarding the relationship between conditional probabilities estimation and causal judgements, assuming that, if anything, probability estimation influences causal judgements and not the other way around. This approach is consistent with a rule‐based perspective of contingency learning (see Perales et al., 2017; Perales & Shanks, 2007, for reviews), which assumes that causal ratings are the product of participants storing the experienced cell frequencies and/or conditional probabilities and combining them in a specific manner. However, associative explanations of contingency learning (Allan, 1993; Matute et al., 2019), trough models such as the popular Rescorla and Wagner (1972) model, would assume that causal judgements emerge from the strengthening of associative links between mental representations, similar to those involved in classical conditioning, with no need of encoding specific frequencies of the different types of events or conditional probabilities of the outcome. From this perspective, estimations of cell frequencies or of conditional probabilities by participants could be inferred from such learned associations (e.g. Vadillo & Luque, 2013; Vadillo & Matute, 2007). The correlational nature of our study does not allow to elucidate whether causal ratings are in fact emerging from the estimated conditional probabilities.

However, previous effective de‐biasing efforts are consistent with the key causal role of giving sufficient weight to *P*(*O*|~*C*) as a way of protecting us from causal illusions. In this sense, educational interventions focused on de‐biasing people against such illusions have more or less explicitly focused on training a more balanced consideration of the different pieces of information available when evaluating a causal relationship. We and other authors developed a first intervention a decade ago (Barberia et al., 2013), addressed to adolescents. The intervention involved a short workshop in which participants were induced into a false causal belief, and were subsequently instructed, among other aspects, in the importance of considering not only the *P*(*O*|*C*), but crucially the *P*(*O*|~*C*), when evaluating a potential causal relationship. This workshop led participants to develop less intense causal illusions in an active contingency learning task that they completed afterwards, and this effect seemed partially mediated by changes in their search strategies (i.e. a decrease in the number of trials in which they introduced the target cause). We later created a new intervention adapted to undergraduate students, finding results analogous to those observed with adolescents regarding the protective effect of the intervention in the development of new causal illusions, both in active (Barberia et al., 2018; Martínez et al., 2023) and passive contingency learning tasks (Rodríguez‐Ferreiro et al., 2023). In a similar vein, a recent study by Chow et al. (2024) showed that short initial instructions regarding the importance of considering the base rate of recovery (i.e. chances of recovery without the treatment) for evaluating a treatment decreased causal illusions regarding the efficacy of an ineffective treatment.

As for limitations, note that our experiments employed unidirectional scales for collecting causal ratings, that is, participants were asked to evaluate the influence of the target drug over the disease on a scale ranging from 0 (*no influence*) to 100 (*total generative influence*). Although unidirectional scales have been habitually used in contemporary studies on causal illusions (e.g. Barberia et al., 2019; Moreno‐Fernández et al., 2021; Vicente et al., 2023), Ng et al. (2024) recently showed that these scales, compared to their bidirectional analogues, can inflate the magnitude of the observed illusions. In our medical scenario, a bidirectional scale might involve values ranging from −100 to +100, the negative ones indicating a preventive relationship (i.e. a harmful influence of the drug over the disease). Future studies could investigate whether the reported pattern of results remains when bidirectional rating scales are introduced.

Furthermore, although in the three studies we included trial‐by‐trial predictions in order to promote the participants sustained attention, our design does not allow to identify potential individual failures to engage with the experiment at some point during the tasks. We reckon that this is less of a problem in the case of the first two studies, which were conducted in person with the presence of an experimenter and, in our view, the replication of the main findings through the three studies reinforces the robustness of the identified effects. In any case, further research could consider including attention checks to try to identify participants not completing the task adequately.

It is also interesting to mention that the percentage of participants assigned to the adjusting cluster was higher in the re‐analysis study (41.3%), compared to the replication (29.3%) and pre‐registered (23%) studies. To this respect, one main difference between these studies was the duration of the training to which the participants were exposed. While the original study by Barberia et al. (2019) was composed of six learning blocks of 48 trials each, both the replication and the pre‐registered studies comprised only three learning blocks of the same length. Hence, it is possible that clusters reflect unequal learning rates (i.e. the persistent cluster would capture those participants that learn at a slower pace) and that a longer training period in the latter two studies might have resulted in a higher proportion of participants being categorized as adjusting rather than persistent. Although this research does not allow to clarify this issue, future studies could investigate this possibility by randomly assigning participants to different conditions based on the length of training.

The application of longitudinal *k*‐means clustering in our study has provided valuable insights into the trajectory patterns of causal illusion ratings over time. One of the primary advantages of this non‐parametric method is its flexibility in handling a variety of trajectory shapes without imposing strict parametric assumptions (Genolini & Falissard, 2010). This flexibility is particularly beneficial in exploratory analyses where the underlying patterns may be complex or non‐linear. Moreover, longitudinal *k*‐means has demonstrated a performance comparable to that of parametric model‐based methods in terms of clustering accuracy (Genolini & Falissard, 2010). However, it is important to acknowledge that while longitudinal *k*‐means performs well in many contexts, its efficacy has not been extensively tested across a wide variety of scenarios, particularly those involving data from experimental paradigms in psychological settings. The specific properties of the temporal distribution of causal ratings may present unique challenges that have not been fully explored. Therefore, further studies are needed to evaluate how well longitudinal *k*‐means performs relative to other methods in these contexts. Furthermore, determining the optimal number of clusters remains a common challenge with clustering algorithms, and longitudinal *k*‐means is no exception. While criteria like the Calinski and Harabasz index provide strong guidance, they do not always yield definitive answers, which can affect the interpretability of the clustering results. In the case of the current research, we replicated our findings across three different studies (including a pre‐registered one), reinforcing the robustness and reliability of the clusters identified. In any case, we encourage future research to compare the performance of longitudinal *k*‐means with other clustering methods, especially in the context of psychological experiments where the temporal distribution of variables, like causal ratings, may exhibit complex patterns. Such comparative analyses would enhance our understanding of the method's strengths and limitations, ultimately informing its appropriate application in various research settings.

This work contributes to the characterization of those individuals that might be particularly sensitive to causal illusions, suggesting that the underweighting of the base rate of the outcome might be a key factor which expresses in encoding, integration and search biases. Interestingly, our data show that these three biases are associated and might all three contribute to the increased sensitivity of some individuals to develop and persevere on causal illusions. These illusions have been pointed out as a possible cognitive facilitator of the development of unfounded beliefs in real life, such as those related with the paranormal (Blanco et al., 2015) or pseudoscience (Torres et al., 2020). Our study deepens our understanding of the development and maintenance of these phenomena, by providing insight on the key differences between persisting individuals and those who are able to adjust their beliefs.

## AUTHOR CONTRIBUTIONS

**J. García‐Arch:** Conceptualization; visualization; formal analysis; writing – original draft; writing – review and editing; methodology; data curation. **J. Rodríguez‐Ferreiro:** Conceptualization; investigation; funding acquisition; writing – review and editing; project administration; supervision. **I. Barberia:** Conceptualization; investigation; funding acquisition; writing – original draft; writing – review and editing; methodology; project administration; supervision.

## FUNDING INFORMATION

This study was supported by grant PID2022‐138016NB‐I00 funded by MICIU/AEI/10.13039/501100011033, FEDER/UE as well as 2021 SGR 01102 by the Agència de Gestió d'Ajuts Universitaris i de Recerca (AGAUR).

## CONFLICT OF INTEREST STATEMENT

Authors declare no conflict of interest.

## Supporting information


Data S1.

## Data Availability

All data, study materials and code associated with this study are openly accessible at https://osf.io/tmc48/?view_only=. This research includes a re‐analysis of existing data, a replication study and a pre‐registered study (https://aspredicted.org/hd56s.pdf).

## Footnotes

^1^
This study was performed with psychology undergraduate students, during a regular class of a course on thinking and problem solving. For simplicity, the method and results of this replication study are presented as Supporting Information (Replication study, Supporting Information).

## References

[bjop12754-bib-0001] Allan, L. G. (1980). A note on measurement of contingency between two binary variables in judgment tasks. Bulletin of the Psychonomic Society, 15(3), 147–149. 10.3758/BF03334492

[bjop12754-bib-0002] Allan, L. G. (1993). Human contingency judgments: Rule based or associative? Psychological Bulletin, 114(3), 435–448. 10.1037/0033-2909.114.3.435 8272465

[bjop12754-bib-0003] Allan, L. G. , & Jenkins, H. M. (1983). The effect of representations of binary variables on judgment of influence. Learning and Motivation, 14(4), 381–405. 10.1016/0023-9690(83)90024-3

[bjop12754-bib-0005] Alloy, L. B. , & Abramson, L. Y. (1979). Judgment of contingency in depressed and nondepressed students: Sadder but wiser? Journal of Experimental Psychology: General, 108(4), 441–485. 10.1037/0096-3445.108.4.441 528910

[bjop12754-bib-0007] Barberia, I. , Blanco, F. , Cubillas, C. P. , & Matute, H. (2013). Implementation and assessment of an intervention to Debias adolescents against causal illusions. PLoS One, 8(8), e71303. 10.1371/journal.pone.0071303 23967189 PMC3743900

[bjop12754-bib-0008] Barberia, I. , Tubau, E. , Matute, H. , & Rodríguez‐Ferreiro, J. (2018). A short educational intervention diminishes causal illusions and specific paranormal beliefs in undergraduates. PLoS One, 13(1), e0191907. 10.1371/journal.pone.0191907 29385184 PMC5792014

[bjop12754-bib-0009] Barberia, I. , Vadillo, M. A. , & Rodríguez‐Ferreiro, J. (2019). Persistence of causal illusions after extensive training. Frontiers in Psychology, 10, 24. 10.3389/fpsyg.2019.00024 30733692 PMC6353834

[bjop12754-bib-0010] Béghin, G. , Gagnon‐St‐Pierre, É. , & Markovits, H. (2021). A dual strategy account of individual differences in information processing in contingency judgments. Journal of Cognitive Psychology, 33(4), 470–481. 10.1080/20445911.2021.1900200

[bjop12754-bib-0011] Béghin, G. , & Markovits, H. (2022). Reasoning strategies and prior knowledge effects in contingency learning. Memory & Cognition, 50(6), 1269–1283. 10.3758/s13421-022-01319-w 35484431

[bjop12754-bib-0012] Blanco, F. , Barberia, I. , & Matute, H. (2015). Individuals who believe in the paranormal expose themselves to biased information and develop more causal illusions than nonbelievers in the laboratory. PLoS One, 10(7), e0131378. 10.1371/journal.pone.0131378 26177025 PMC4503786

[bjop12754-bib-0014] Blanco, F. , Matute, H. , & Vadillo, M. A. (2011). Making the uncontrollable seem controllable: The role of action in the illusion of control. Quarterly Journal of Experimental Psychology, 64(7), 1290–1304. 10.1080/17470218.2011.552727 21432736

[bjop12754-bib-0016] Blanco, F. , Matute, H. , & Vadillo, M. A. (2013). Interactive effects of the probability of the cue and the probability of the outcome on the overestimation of null contingency. Learning and Behavior, 41(4), 333–340. 10.3758/s13420-013-0108-8 23529636

[bjop12754-bib-0017] Blanco, F. , Moreno‐Fernández, M. M. , & Matute, H. (2020). Are the symptoms really remitting? How the subjective interpretation of outcomes can produce an illusion of causality. Judgment and Decision making, 15(4), 572–585. 10.1017/S1930297500007506

[bjop12754-bib-0019] Chow, J. Y. , Goldwater, M. B. , Colagiuri, B. , & Livesey, E. J. (2024). Instruction on the scientific method provides (some) protection against illusions of causality. Open Mind, 8, 639–665. 10.1162/opmi_a_00141 38828432 PMC11142631

[bjop12754-bib-0020] Chow, J. Y. L. , Colagiuri, B. , & Livesey, E. J. (2019). Bridging the divide between causal illusions in the laboratory and the real world: The effects of outcome density with a variable continuous outcome. Cognitive Research: Principles and Implications, 4(1), 1. 10.1186/s41235-018-0149-9 30693393 PMC6352562

[bjop12754-bib-0021] Genolini, C. , & Falissard, B. (2010). KmL: k‐means for longitudinal data. Computational Statistics, 25(2), 317–328. 10.1007/s00180-009-0178-4

[bjop12754-bib-0022] Genolini, C. , & Falissard, B. (2011). KmL: A package to cluster longitudinal data. Computer Methods and Programs in Biomedicine, 104(3), e112–e121. 10.1016/j.cmpb.2011.05.008 21708413

[bjop12754-bib-0023] Genolini, C. , & Falissard, B. (2016). Package “kml.”. https://cran.r‐project.org/web/packages/kml/kml.pdf (24 September 2023, date last accessed).

[bjop12754-bib-0024] Griffiths, O. , Shehabi, N. , Murphy, R. A. , & Le Pelley, M. E. (2019). Superstition predicts perception of illusory control. British Journal of Psychology, 110(3), 499–518. 10.1111/bjop.12344 30144046

[bjop12754-bib-0025] Hall, M. H. , Holton, K. , Chittenden, T. , Ongur, D. , Eklund, K. , Montrose, D. , & Keshavan, M. (2017). 486. Longitudinal recovery trajectories of patients with first episode psychosis. Biological Psychiatry, 81(10), S198. 10.1016/j.biopsych.2017.02.970

[bjop12754-bib-0026] Hannah, S. D. , & Beneteau, J. L. (2009). Just tell me what to do: Bringing back experimenter control in active contingency tasks with the command‐performance procedure and finding cue density effects along the way. Canadian Journal of Experimental Psychology, 63(1), 59–73. 10.1037/a0013403 19271817

[bjop12754-bib-0027] Lenth, R. (2020). Emmeans: estimated marginal means, aka least‐squares means. R package version 1.4.7. https://CRAN.R‐project.org/package=emmeans

[bjop12754-bib-0029] Lovibond, P. , Chow, J. Y. , & Lee, J. C. (2023). How do participants interpret trials from individual cells in a causal illusion task? Proceedings of the annual meeting of the cognitive science society, 45. https://escholarship.org/uc/item/6z13c6d6

[bjop12754-bib-0060] Mandel, D. R. , & Lehman, D. R. (1998). Integration of contingency information in judgments of cause, covariation, and probability. Journal of Experimental Psychology: General, 127(3), 269–285. https://doi‐org.sire.ub.edu/10.1037/0096‐3445.127.3.269

[bjop12754-bib-0030] Martínez, N. , Rodríguez‐Ferreiro, J. , Barberia, I. , & Matute, H. (2023). A debiasing intervention to reduce the causality bias in undergraduates: The role of a bias induction phase. Current Psychology, 42(36), 32456–32468. 10.1007/s12144-022-04197-2

[bjop12754-bib-0031] Matute, H. , Blanco, F. , & Díaz‐Lago, M. (2019). Learning mechanisms underlying accurate and biased contingency judgments. Journal of Experimental Psychology. Animal Learning and Cognition, 45(4), 373–389. 10.1037/xan0000222 31380677

[bjop12754-bib-0032] Matute, H. , Blanco, F. , Yarritu, I. , Díaz‐Lago, M. , Vadillo, M. A. , & Barberia, I. (2015). Illusions of causality: How they bias our everyday thinking and how they could be reduced. Frontiers in Psychology, 6, 888. 10.3389/fpsyg.2015.00888 26191014 PMC4488611

[bjop12754-bib-0033] Matute, H. , Yarritu, I. , & Vadillo, M. A. (2011). Illusions of causality at the heart of pseudoscience. British Journal of Psychology, 102(3), 392–405. 10.1348/000712610X532210 21751996

[bjop12754-bib-0034] Moreno‐Fernández, M. M. , Blanco, F. , & Matute, H. (2021). The tendency to stop collecting information is linked to illusions of causality. Scientific Reports, 11(1), 3942. 10.1038/s41598-021-82075-w 33594129 PMC7887230

[bjop12754-bib-0035] Neath, A. A. , & Cavanaugh, J. E. (2012). The Bayesian information criterion: Background, derivation, and applications. Wiley Interdisciplinary Reviews: Computational Statistics, 4(2), 199–203. 10.1002/wics.199

[bjop12754-bib-0036] Ng, D. W. , Lee, J. C. , & Lovibond, P. F. (2024). Unidirectional rating scales overestimate the illusory causation phenomenon. Quarterly Journal of Experimental Psychology, 77(3), 551–562. 10.1177/17470218231175003 PMC10880420 37114953

[bjop12754-bib-0038] Panlilio, L. V. , Stull, S. W. , Bertz, J. W. , Burgess‐Hull, A. J. , Kowalczyk, W. J. , Phillips, K. A. , Epstein, D. H. , & Preston, K. L. (2020). Beyond abstinence and relapse: Cluster analysis of drug‐use patterns during treatment as an outcome measure for clinical trials. Psychopharmacology, 237(11), 3369–3381. 10.1007/s00213-020-05618-5 32990768 PMC7579498

[bjop12754-bib-0039] Perales, J. C. , Catena, A. , Cándido, A. , & Maldonado, A. (2017). Rules of causal judgment: Mapping statistical information onto causal beliefs. In M. R. Waldmann (Ed.), Oxford handbook of causal reasoning (Vol. 1, pp. 29–51). Oxford University Press.

[bjop12754-bib-0040] Perales, J. C. , & Shanks, D. R. (2007). Models of covariation‐based causal judgment: A review and synthesis. Psychonomic Bulletin and Review, 14(4), 577–596. 10.3758/BF03196807 17972719

[bjop12754-bib-0041] Rescorla, R. A. , & Wagner, A. R. (1972). A theory of Pavlovian conditioning: Variations in the effectiveness of reinforcement and nonreinforcement. In A. H. Black & W. F. Prokasy (Eds.), Classical conditioning II: Current research and theory (pp. 64–99). Appleton‐Century Crofts.

[bjop12754-bib-0042] Rodríguez‐Ferreiro, J. , Vadillo, M. A. , & Barberia, I. (2023). Debiasing causal inferences: Over and beyond suboptimal sampling. Teaching of Psychology, 50(3), 230–236. 10.1177/00986283211048394

[bjop12754-bib-0043] Torres, M. N. , Barberia, I. , & Rodríguez‐Ferreiro, J. (2020). Causal illusion as a cognitive basis of pseudoscientific beliefs. British Journal of Psychology, 111(4), 840–852. 10.1111/bjop.12441 32040216

[bjop12754-bib-0044] Torres, M. N. , Barberia, I. , & Rodríguez‐Ferreiro, J. (2022). Causal illusion in the core of pseudoscientific beliefs: The role of information interpretation and search strategies. PLoS One, 17(9), e0272201. 10.1371/journal.pone.0272201 36084028 PMC9462769

[bjop12754-bib-0046] Vadillo, M. A. , & Luque, D. (2013). Dissociations among judgments do not reflect cognitive priority: An associative explanation of memory for frequency information in contingency learning. Canadian Journal of Experimental Psychology/Revue Canadienne de Psychologie expérimentale, 67(1), 60–71. 10.1037/a0027617 22506878

[bjop12754-bib-0047] Vadillo, M. A. , & Matute, H. (2007). Predictions and causal estimations are not supported by the same associative structure. Quarterly Journal of Experimental Psychology, 60(3), 433–447. 10.1080/17470210601002520 17366310

[bjop12754-bib-0048] Vicente, L. , Blanco, F. , & Matute, H. (2023). I want to believe: Prior beliefs influence judgments about the effectiveness of both alternative and scientific medicine. Judgment and Decision making, 18, E1. 10.1017/jdm.2022.3

[bjop12754-bib-0050] Wood, M. E. , Lupattelli, A. , Palmsten, K. , Bandoli, G. , Hurault‐Delarue, C. , Damase‐Michel, C. , Chambers, C. D. , Nordeng, H. , & van Gelder, M. M. (2021). Longitudinal methods for modeling exposures in pharmacoepidemiologic studies in pregnancy. Epidemiologic Reviews, 43(1), 130–146. 10.1093/epirev/mxab002 PMC8763114 34100086

[bjop12754-bib-0051] Yarritu, I. , Matute, H. , & Vadillo, M. A. (2014). Illusion of control: The role of personal involvement. Experimental Psychology, 61(1), 38–47. 10.1027/1618-3169/a000225 23948387 PMC4013923

