# Supplementary material for: Individual differences in the evolution of causal illusions
Source: Br J Psychol. 2024 Dec 6;116(2):336–53. doi: 10.1111/bjop.12754 (PMC11984348; doi:10.1111/bjop.12754)
Supplement: Supplementary file 1 — Data S1. [file BJOP-116-336-s001.docx]

**Supplementary Information**

| **Table S1**. Comparison between groups (causal ratings) in each time-point | | | | | | |
| --- | --- | --- | --- | --- | --- | --- |
| *contrast* | *Time* | *dif* | *SE* | *df* | *t* | *p* |
|  | 1 | 18.488 | 5.102 | 73 | 3.624 | .001 |
|  | 2 | 30.982 | 5.000 | 73 | 6.196 | < .001 |
| Persistent - Adjusting | 3 | 39.663 | 3.900 | 73 | 10.171 | < .001 |
|  | 4 | 48.856 | 3.939 | 73 | 12.402 | < .001 |
|  | 5 | 49.403 | 3.996 | 73 | 12.363 | < .001 |
|  | 6 | 51.080 | 4.256 | 73 | 12.003 | < .001 |

**Cluster differences in estimations of conditional probabilities (Reanalysis)**

Once identified the differences in cluster trajectories, we analysed whether clusters also differed in their subjective estimations of the conditional probabilities (Figure 1, A). We conducted two separate mixed ANOVAs, one for each subjective probability estimation. Results suggested that clusters significantly differed in the *P*(O│C)_subj_, *F*(1,73) = 5.776, *p* = 0.019, *η^2^* = 0.073, 90% CI[0.064, 0.078]. No significant effects were found for Time, *F*(4.069,297.049) = 1.086, *p* = 0.364, *η^2^* = 0.015 90% CI[0.002, 0.005] or Cluster x Time interaction, *F*(4.069,297.049) = 1.179, *p* = 0.32, *η^2^* = 0.016, 90% CI[0.004, 0.007]. Post-hoc analysis showed that the effect of Cluster was driven by a higher *P*(O│C)_subj_ in the *persistent* than in the *adjusting* cluster (*M* = 7.008, *SE* = 2.921, t(73) = 2.403, *d* = 0.562, 90% CI[0.168, 0.953]). Results regarding *P*(O│~C)_subj_ showed a significant main effect of Cluster, *F*(1,73) = 6.334, *p* = 0.014, *η*^2^ = 0.08, 90% CI[0.07, 0.085], a significant main effect of Time, *F*(3.806,277.825) = 6.971, p <.001, *η^2^* = 0.087, 90% *CI*[0.073, 0.081], and a non-significant Cluster x Time interaction, *F*(3.806,277.825) = 2.263, *p* = 0.066, *η*² = 0.03, 90% CI[0.018, 0.022].

| **Table S2.** Mixed-effects models predicting causal ratings, and associated BICs |  |
| --- | --- |
| Mixed-effects models (formula) | BIC |
| Causal rating ~ (Time) * Cluster + (1 \| Participant) | 3886.59 |
| Causal rating ~ [Time + *P*(O│~C)_subj_] * Cluster + (1 \| Participant) | 3864.81 |
| Causal rating ~ Time + *P*(O│~C)_subj_ + Cluster + (1 \| Participant) | 3913.31 |
| Causal rating ~ Time + *P*(O│C)_subj_ + Cluster + (1 \| Participant) | 3867.82 |
| Causal rating ~ Time + *P*(O│C)_subj_ + *P*(O│~C)_subj_ + Cluster + (1 \| Participant) | 3810.24 |
| Causal rating ~ [Time + *P*(O│~C)_subj_] * Cluster + (1 \| Participant) | 3864.40 |
| Causal rating ~ [Time + *P*(O│C)_subj_] * Cluster + (1 \| Participant) | 3795.40 |
| **Causal rating ~ [Time + *P*(O│C)_subj_ + *P*(O│~C)_subj_] * Cluster + (1 \| Participant)** | **3728.36** |
| Causal rating ~ [*P*(O│C)_subj_ + *P*(O│~C)_subj_] * Cluster * Time + (1 \| Participant) | 3865.17 |

| **Table S3.** Global tests on models’ predictors (Satterthwaite's method) | | | | |
| --- | --- | --- | --- | --- |
| *Parameter* | *Df_numerator_* | *Df_denominator_* | *F* | *p* |
| Time | 5 | 357.96 | 4.74 | p <.001 |
| *P*(O│C)_subj_ | 1 | 409.91 | 178.74 | p <.001 |
| *P*(O│~C)_subj_ | 1 | 390.48 | 79.64 | p <.001 |
| Cluster | 1 | 337.01 | 9.28 | .003 |
| Time * Cluster | 5 | 357.96 | 6.81 | p <.001 |
| *P*(O│C)_subj_ * Cluster | 1 | 409.91 | 10.92 | .001 |
| *P*(O│~C)_subj_ * Cluster | 1 | 390.48 | 39.23 | p <.001 |

**Replication Study:**

In this replication study, we aimed to test the robustness of the results encountered during the reanalysis of Barberia et al. (2019). The new study was similar to that by Barberia et al. (2019, Recurrent group), except for the following minor changes. First, given that the learning pattern of the different clusters (*persistent* and *adjusting*) was already apparent after the third measure^[[1]](#footnote-1)^, we considered that three blocks of 48 trials would be sufficient to replicate this effect. We also included a categorical yes/no question (i.e., “In short, would you say that Batatrim [the medicine] is effective or not?”) after the causal rating questions of the first and third blocks intended as exploratory measures of the qualitative interpretation of the numerical causal ratings provided by the participants.

**Method**

**Participants**

A group of 181 volunteers, 159 females, *M_age_* = 21.75, *SD_age_* = 3.13, took part in the study. They were all Psychology undergraduate students, who completed the contingency learning task at the beginning of a lecture about causal detection. The study protocol, which included informed consent for the data to be used with research purposes, was approved by the ethics committee of the University of Barcelona (Institutional Review Board IRB00003099).

**Procedure**

The task was programmed and presented through Qualtrics and it followed the procedure applied by Barberia et al. (2019). In brief, the participants were told that they were going to be presented with medical records of several patients suffering from a dangerous disease called Lindsay Syndrome which causes life-threatening crises (e.g., Blanco et al., 2011, 2013; Matute et al., 2011). Some of the patients, all of which would be suffering from a crisis episode, would receive the experimental drug “Batatrim” while others would not, and, for each of them, the participants would be informed whether the patient recovered or not. The goal of the task was to discover whether the drug was effective as a treatment for the disease.

The 144 patients were presented pseudorandomly in three blocks of 48 so that, in each block, the frequencies of patients receiving or not receiving the drug, and overcoming or not the crisis was that indicated in Table 1 (see *Manuscript*). After each block, the volunteers had to respond to the following questions: 1) causal rating - “To what extent do you think that Batatrim is effective in overcoming the crises produced by the Lindsay Syndrome?” (from 0 - not effective at all, to 100 - totally effective); 2) *P*(O│C)_subj_ - “Imagine 100 NEW PATIENTS that are suffering a crisis produced by the Lindsay Syndrome and TAKE BATATRIM. How many of them do you think will overcome the crisis?”; 3) *P*(O│~C)_subj_ - “Imagine 100 NEW PATIENTS that are suffering a crisis produced by the Lindsay Syndrome and DO NOT TAKE ANYTHING. How many of them do you think will overcome the crisis?”. The order of presentation of the questions was counterbalanced between participants, but kept constant for each of them throughout the blocks. Different to the original procedure, we also included a categorical yes/no question (i.e., In short, would you say that Batatrim is effective or not?) after the causal rating questions of the first and third blocks. Moreover, while in Barberia et al. (2019) participants were instructed to take short rests whenever they needed it, this aspect of the instructions was eliminated for the present study.

The study, based on a convenience sample, was not preregistered, nor was the sample size determined in advance. All data, analysis code, and research materials are available at [https://osf.io/tmc48/?view_only=3db055102a8641ed9acaaa452db39a4e]. Data were analyzed using R, version 4.0.0 (R Core Team, 2020).

**Results**

Consistent with our previous findings, the Kml algorithm found 2 clusters as the best possible partition. 70.7% of the participants were identified as belonging to cluster A and 29.3% to cluster B. We subsequently aimed to replicate our findings in the reanalysis by Barberia et al. (2019). We expected to find differences in cluster trajectories representing a differential evolution of null contingency learning, with only one cluster displaying a reduction of the causal illusion over repeated exposures. As expected, we found that cluster A was characterized by a stable pattern of causal ratings, suggesting no change across time (*persistent* cluster). Cluster B (*adjusting* cluster) showed a decrease in their causal illusions across time (Figure S1).


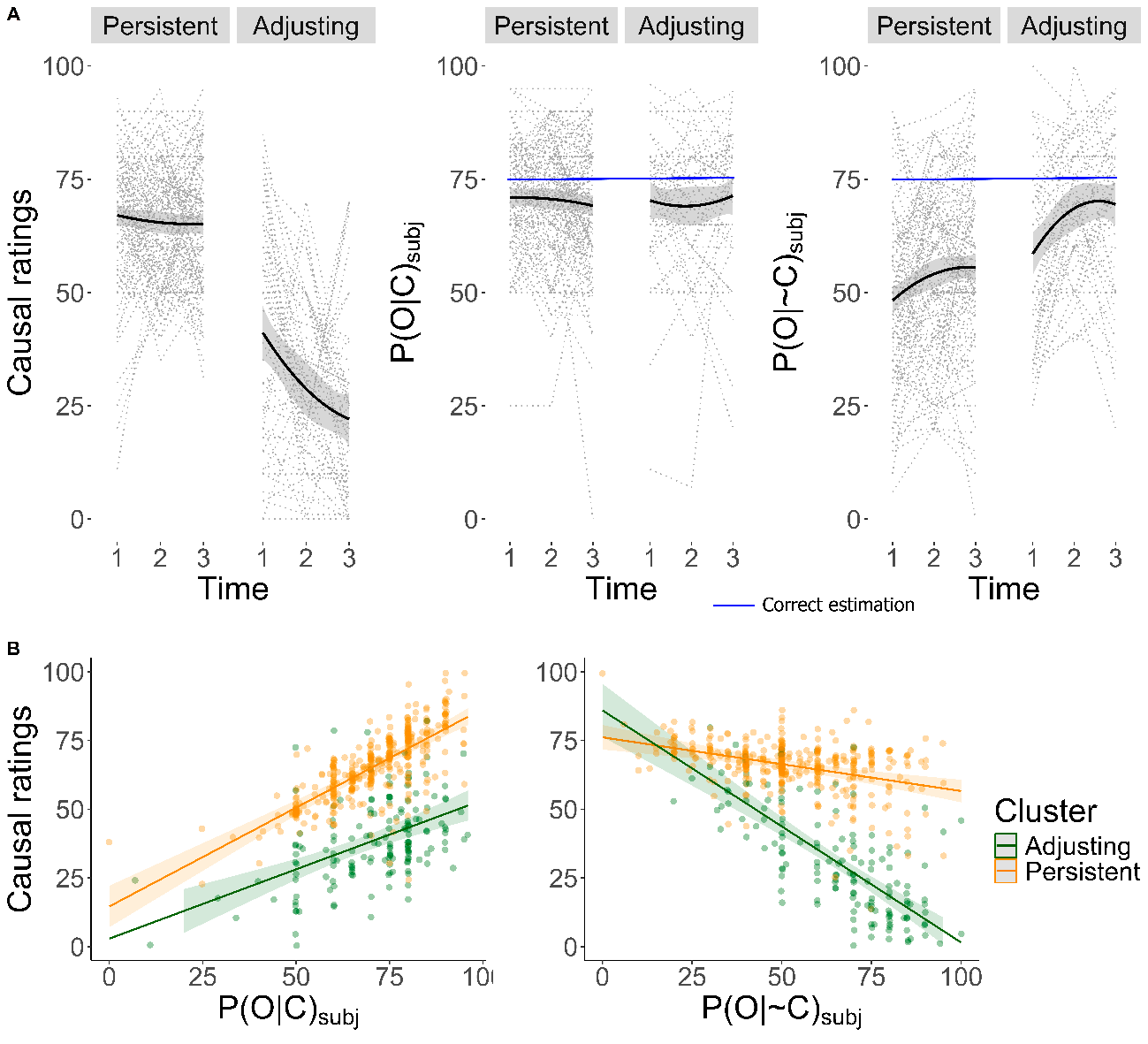


**Figure S1.** Distribution of Causal illusion ratings, P(O|C)subj (B), and P(O│~C)subj over time as a function of cluster membership in our replication study (Panel A). Relationship of P(O|C)subj (A) and P(O|~C)subj (B) with causal ratings by cluster membership (Panel B).

To test the observed differences, we run a mixed ANOVA with causal ratings as the dependent variable, Cluster as a between-subjects factor and Time as a within-subjects’ factor. Results showed a significant main effect of Cluster *F*(1,179) = 353.713, *p* < .001, *η*^2^ = 0.664, 90% CI[.657, .665] a significant main effect of Time *F*(1.879,336.338) = 20.007, *p* = 0, *η*^2^ = .101, 90% CI[.094, .101], and a significant Cluster x Time interaction *F*(1.879,336.338) = 13.085, *p* <.001, *η*^2^ = 0.068, 90% CI[.062, .068]. Post-hoc pairwise comparisons revealed that participants in the *adjusting* cluster displayed significantly less causal illusion in all time points than those in the *persistent* cluster, minimum *t*(179)= 8.631, p < .001). Results from a polynomial trend contrast showed no significant variation in causal ratings across Time for the *persistent* cluster (linear: *M* = -1.95, *SE* = 2.05, *t*(179) = -.954, *p* = .341, quadratic: *M* = 1.23, *SE* = 4.26, *t*(179) = .451, *p* = .652). The analysis also showed that the best trend to characterize the causal illusion decrease in the *adjusting* cluster was linear *M* = -19.06, *SE* = 3.18, t(179) = -5.989, *p* <.001 (quadratic: *M* = 5.66 *SE* = 4.26, *t*(179) = 1.330, *p* = .185).

Subsequently, we analysed whether clusters also differed in their subjective estimations of conditional probabilities. We conducted two separate mixed ANOVAs, one for each subjective probability estimation. Results regarding *P*(O│C)_subj_ returned no significant differences between clusters *F*(1,179) < .001, *p* = .989, *η^2^* < .001, 90% CI[< .001, < .001]. Moreover, no significant effects were found for Time *F*(1.836,328.647) = .189, *p* = .809, *η^2^* = .001, 90% CI[<.001, .002] or Cluster x Time interaction *F*(1.836,328.647) = 1.228, *p* = .292, *η^2^* = .007, 90% CI[0.003, 0.005] (Figure S1, B). Results regarding *P*(O│~C) _subj_ showed a significant main effect of Cluster *F*(1, 179) = 30.427, *p* < .001, a significant main effect of Time *F*(2, 358) = 22.114, *p* <.001, and no significant Cluster x Time interaction *F*(2, 358) = 1.181, *p* =.308. Post-hoc contrast revealed that the *persistent* cluster provided a significantly lower *P*(O│~C)_subj_ than the *adjusting* cluster (*M* = -12.889, *SE* = 2.341, *t.ratio*(179) = -5.516, *p* <.001, *d* = .82). Post-hoc analysis of the Time effect revealed that the *P*(O│~C)_subj_ was lower in Time 1 with respect to Time 2 and Time 3 [*M* = -7.966, *SE* = 1.490, *t.ratio*(179) = -5.347, *p* <.001, *d* = .799; *M* = -9.071, *SE* = 1.490, *t.ratio*(179) = -5.433, *p* <.001, *d* = .892]. No significant differences were found between Time 2 and Time 3 [*M* = -1.111, *SE* = 1.28, *t.ratio*(179) = -.869, p = .661, *d* = .129].

Next, we aimed to replicate the model obtained for the prediction of causal ratings in the reanalysed data by Barberia et al. (2019). To that end, we tested whether the predictive capacity of the *P*(O│C)_subj_ and *P*(O│~C)_subj_ over causal ratings was different between clusters (and Time). In concordance with this goal, we built a linear mixed effects model with causal ratings as the dependent variable, and Cluster, Time, *P*(O│C)_subj_, *P*(O│~C)_subj_, and all Cluster two-way interactions as fixed effects. As in the previous reanalysis of existing data, we constructed 8 additional models to test if any of them accounted for causal ratings better than the hypothesized model. We compared the fitted models by means of the Bayesian Information Criteria index (BIC). The selected model replicated that obtained in the reanalysis (i.e., Illusion ~ [Time + *P*(O│C)_subj_ + *P*(O│~C)_subj_] * Cluster + [1 | Participant]). Global tests on model predictors returned statistically significant effects for all model parameters (all *p* <.03). We subsequently decomposed both target interactions *P*(O│C) _subj_ x Cluster and *P*(O│~C) _subj_ x Cluster to explore whether results matched those obtained before. Post-hoc analyses showed that both clusters displayed a negative relationship between *P*(O│~C)_subj_ and causal ratings, and that the interaction effect Cluster x *P*(O│~C) _subj_ was due to a greater negative slope in the *adjusting* cluster as compared to the *persistent* cluster (*persistent*: *β* = -.195, SE = .041, 95% CI[-.275, -.116], *adjusting*: *β* = -.844, SE = .072, 95% CI[-.987, -.702]). Post-hoc analyses also revealed that both clusters displayed a positive relationship between *P*(O│C)_subj_ and causal ratings and that the interaction effect Cluster x *P*(O│C)_subj_ was due to a greater positive slope in the *persistent* cluster as compared the *adjusting* cluster (*persistent*: *β* = .719, SE = .053, 95% CI[.614, .824], *adjusting*: *β* = .505, SE = .082, 95% CI[.343, .666]), which fully replicated the results obtained in the reanalysis (see Figure S1, B). As in the reanalysis, we also tested if a model including the difference between *P*(O│C)_subj_ and *P*(O│~C)_subj_ instead of *P*(O│C)_subj_ and *P*(O│~C)_subj_ separately would improve the current model’s fit to the data. Results showed that the model including separate components for *P*(O│C)_subj_ and *P*(O│~C)_subj_ outperformed the new model tested (BIC1: 4376.906; BIC2: 4440.622, respectively).

**Initial written instructions: Preregistered study.**

You are going to participate in an experiment. Please stay focused on the task until its completion. You should not take notes on the information we present to you. Click on the arrow to continue

Imagine that you are a doctor working in a research laboratory at a university. You are a specialist in a very rare and dangerous disease called "Lindsay Syndrome", which must be treated very quickly in the emergency room. The crises caused by this disease could be cured immediately with a drug called "Batatrim", but this drug is still in an experimental phase, so its effectiveness has not yet been clearly proven.

Next, we are going to present a series of medical records of patients who are experiencing a Lindsay Syndrome crisis. In each record you will see a patient and we will tell you whether or not we have administered Batatrim to the patient. You must indicate whether you think the patient will overcome the crisis or not. Once you have made your prediction, we will tell you if the patient overcame the crisis. Next you will observe the following patient.

TRY TO FIND OUT HOW EFFECTIVE BATATRIM IS.

When you have observed a good number of patients, we will ask you some questions.

**Cluster differences in causal ratings (Preregistered study)**

Post-hoc pairwise comparisons revealed that participants in the adjusting cluster displayed significantly less causal illusion in all time points than those in the persistent cluster (minimum *t*(298) = 14.057, *p* < .001). Results from a polynomial trend contrast showed no significant variation in causal ratings across Time for the persistent cluster (linear: *M* = -1.35, *SE* = 1.52, *t*(298) = -.886, *p* = .611, quadratic: *M* = -4.19, *SE* = 2.06, *t*(298) = -2.031, *p* = .084). The analysis also showed that the best trend to characterize the causal illusion decrease in the adjusting cluster was linear *M* = -13.54, *SE* = 2.79, *t*(298) = -4.855, *p* <.001 (quadratic: *M* = 5.51 *SE* = 3.77, *t*(298) = 1.459, *p* = .271).

1. In order to ensure that we could reduce the task to three blocks, we conducted a mixed ANOVA including causal ratings as a dependent variable, Time as a within-subjects factor and Cluster as a between subjects factor, but limiting Time to blocks 1 to 3. Results showed that the critical Time x Cluster interaction effect was still statistically significant when only the first three blocks of trials were included, *F*(1.79, 130.61) = 8.666, *p* <.001, *η^2^*= .093, 90% CI[.092, .105]. [↑](#footnote-ref-1)
